# Adolescent mental health in sub-Saharan Africa: crisis? What crisis? Solution? What solution?

**DOI:** 10.1080/16549716.2024.2437883

**Published:** 2025-01-17

**Authors:** Claire Hart, Shane A. Norris

**Affiliations:** a SA MRC/Wits Developmental Pathways for Health Research Unit, Department of Paediatrics, Faculty of Health Sciences, School of Clinical Medicine, University of the Witwatersrand, Johannesburg, South Africa; b School of Human Development and Health, University of Southampton, Southampton, UK

**Keywords:** Adolescent mental health, sub-Saharan Africa, low- and middle-income countries (LMICs), stigma, community-based services, access to care

## Abstract

Addressing adolescent mental health care across sub-Saharan Africa faces numerous challenges, including underfunded public health systems, a shortage of mental health professionals, barriers to access, and pervasive stigma. Untreated adolescents often experience worsening symptoms, academic and social difficulties, physical health risks, and engage in risky behaviours. Early detection and appropriate treatment of common mental health conditions can support adolescents in developing robust social and emotional foundations and enhancing their mental well-being. Ensuring adolescents receive the mental health care required for healthy development depends on collaborative, evidence-based solutions that consider the contextual challenges of sub-Saharan Africa. Innovative community-based solutions to mental health services may significantly improve accessibility and support adolescents close to their homes and schools. For example, co-creation and peer-delivered interventions with professional supervision may enhance uptake and reduce stigma. This short article adds to the current debate arguing for working with communities and implementing community mental health services for common mental health conditions. Sensitivity to community-specific challenges and building referral networks are crucial for effective care. Investing in these strategies, alongside increasing mental health literacy, could lead to affordable and significant interventions to address adolescent mental health.

## Paper context

- **Main findings:** Addressing adolescent mental health in sub-Saharan Africa requires collaborative, evidence-based solutions that consider local challenges.
- **Added knowledge:** To improve adolescent mental health care in sub-Saharan Africa, it is crucial to collaborate with communities, develop community-specific solutions, and establish referral networks.
- **Global health impact for policy and action:** Investing in adolescent mental health care and enhancing mental health literacy can lead to significant, cost-effective interventions that support healthy development.

## Background

**Figure 1.**
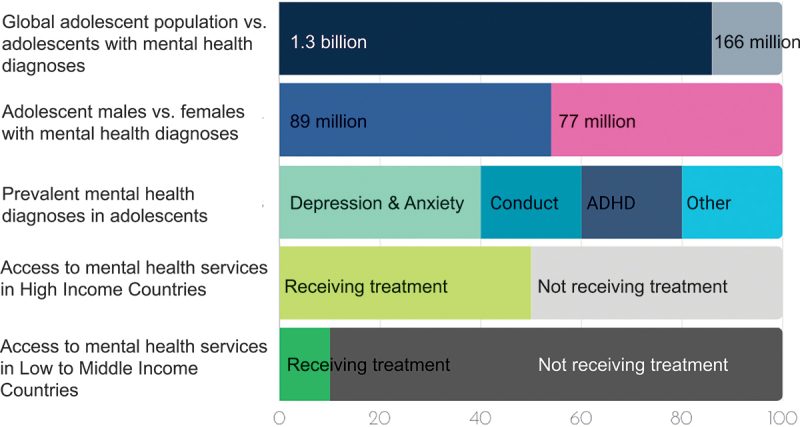
Global overview of adolescent mental health burden in percentages.

Mental health conditions in adolescents constitute a significant burden of disease and disability globally [1–4]. Common mental disorders, such as depression and anxiety, often peak during adolescence, while more severe, chronic mental disorders, such as schizophrenia and bipolar disorder, typically emerge in early adulthood. Adolescents in low- and middle-income countries (LIMCs) are disproportionately affected by common mental health conditions, yet the public health sectors in these regions lack sufficient funding, management, and treatment services for adolescent mental health. Estimates suggest that up to 90% of individuals with mental conditions in these regions do not receive any treatment (Figure 1) [5]. Even in high-income countries this is a problem, where between 35% and 50% of people with mental conditions receive no treatment [6].

**Figure 2.**
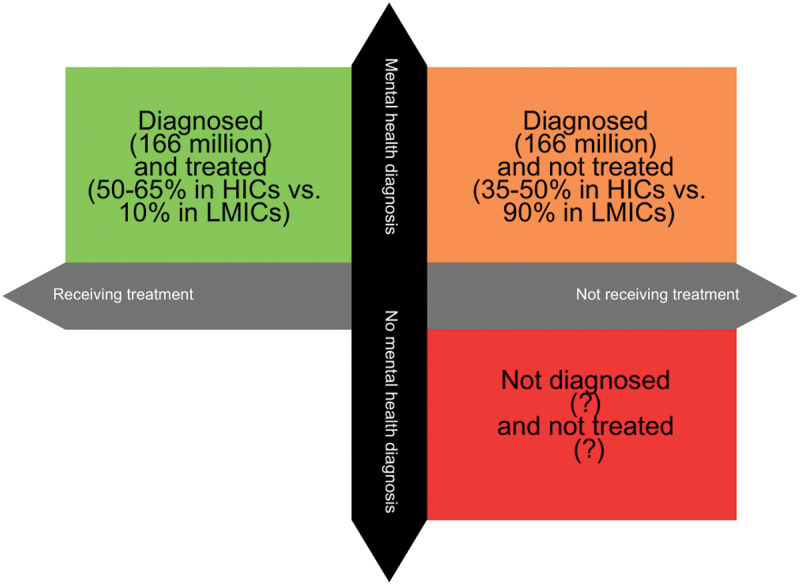
Adolescent mental health treatment gap.

Adolescents who do not receive mental health treatment experience worsening symptoms with long-term debilitating effects. These individuals face persistent academic, family, and social challenges, leading to difficulties in maintaining employment and relationships into adulthood. They are also at increased risk for physical health issues such as cardiovascular disease and weakened immune function [7]. Premature death due to suicide, a leading cause of death in adolescents, is of particular concern [1]. Additionally, self-harm and risky behaviours, including substance abuse, unsafe sexual practices, and reckless driving, negatively impact their mental and physical health [4]. The most vulnerable adolescents are those who remain undiagnosed, those who cannot access mental health care, and those who receive inappropriate or unreliable treatment (see Figure 2).

## Key challenges in adolescent mental health in sub-Saharan Africa

Adolescents in sub-Saharan Africa (SSA), the fastest-growing adolescent population in the world, experience significant mental health challenges [8–10]. Recent reviews revealed a 27% prevalence of depression, 30% prevalence of anxiety disorders, 21% prevalence of post-traumatic stress disorder (PTSD), and 12% prevalence of suicidal ideation for adolescents in the region [10,11]. These figures are notably higher than global prevalence estimates for these conditions: 13% for depression, 7–10% for anxiety, 3–6% for PTSD, and 10% for suicidal ideation [4]. The median annual persistence rate for any mental health disorder is reported at 80.0% [12], and these conditions, which tend to begin in adolescence, require long-term intervention. In SSA, high levels of comorbidity are common, such as HIV/AIDS and tuberculosis [10,13], cardiovascular disease and diabetes mellitus [14], elevated rates of violence and injury [15], and maternal and child illness [16]. Given the compounded burden of mental health disorders and related health issues, urgent and sustained efforts are needed to address the mental health needs of adolescents in SSA, ensuring timely and effective care to improve long-term health and well-being in the region.

**Figure 3.**
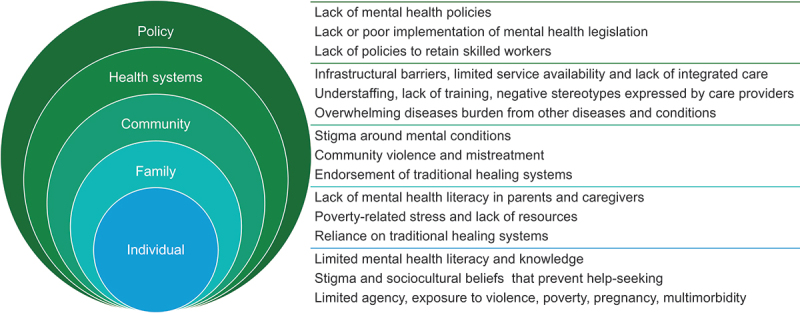
Barriers to mental health adolescent care.

The social-ecological framework for mental health and well-being guided the identification of key barriers to adolescent mental health in SSA (see Figure 3) [10,17]. This framework emphasizes that mental health outcomes are influenced by multiple levels of interaction, including individual, interpersonal, community, and societal factors. This framework is valuable in understanding how to address adolescent mental health challenges as it provides a lens through which to understand the systemic barriers and opportunities for improvement in adolescent mental health care.

At the policy level, adolescent mental health in SSA is frequently overlooked amid numerous other public health challenges. Less than 1% of the annual health budgets are allocated to mental health, and 40% of African countries lack any dedicated budget for mental health [10,11]. This underfunding contributes to widespread undiagnosed and untreated mental health conditions, exacerbating the difficulties faced by adolescents in need of care.

At a health systems level, many adolescents struggle to access necessary mental health care, primarily due to the limited availability of public health services [18]. The UNICEF South Africa U-Report poll showed that 60% of youth needed mental health support over the past year, with many not knowing where to get help [19]. Mental health care in rural areas, along with preventive and promotive aspects of mental health, remains under-resourced and underdeveloped [20]. Situational analyses have also revealed a severe deficit of mental health professionals, with only 0.1 psychiatrists and 0.2 health workers for children and adolescents per 100,000 in the African Region in 2020, which is ten times lower than in most high-income countries [10,21]. In many cases, primary health care workers and non-specialist health workers are compelled to deliver mental health services, despite facing considerable strain due to high caseloads and minimal training in mental health [10,22]. These healthcare workers lack the necessary resources to effectively identify and support young individuals grappling with common mental health issues [23]. This situation often leads to suboptimal care for patients, with paediatrics and maternal and child health departments, schools, social welfare services and facilities, and juvenile justice system and prisons often left to deal with the burden of untreated mental health cases [24].

At a community level, a variety of health and socioeconomic challenges increase adolescents’ vulnerability to mental health conditions. These include poverty, food insecurity, exposure to conflict and violence, education exclusion, unplanned pregnancy, and unemployment. The intersection of these challenges further complicates efforts to effectively prevent, detect early, and treat adolescent depression and anxiety in SSA. Research highlights the pervasive mental health stigma at the community level, with high levels of prejudice against individuals with mental disorders across the region [25]. Adolescents facing mental health challenges often endure social stigma, which can lead to financial difficulties, social isolation, and discrimination [26]. This mental health stigma prevents adolescents from seeking the necessary care and support essential for early detection and treatment of mental health issues. It also undermines community-wide efforts to address mental health issues, creating a cycle of neglect and insufficient care for mental health needs.

## Are community co-created solutions an approach to support adolescent mental health care?

A possible solution to address barriers to mental health care and bridge the adolescent mental health treatment gap in the region is through the co-creation of interventions where evidence-based researchers collaborate with adolescents and other community stakeholders [10,27,28]. This approach emphasizes bringing mental health care directly to communities, particularly in rural and remote areas, in alignment with the community mental health model widely adopted by public health systems both within Africa and globally. Task-sharing, where non-specialist providers are trained to deliver mental health interventions in clinic or community settings, has been a significant development in the region to address the mental health treatment gap. In resource-constrained contexts, community-based services present a critical opportunity to improve access to mental health care. By leveraging existing community resources, these services can enhance both service delivery and training, enabling community health workers to provide care as close as possible to where people live, work, and study. For adolescents, receiving care within their communities increases accessibility and allows them to engage with mental health services without being dependent on their caregivers’ income or work patterns. However, it is crucial to carefully consider the quality of this care to ensure its effectiveness and sensitivity to contextual challenges in the community.

Interventions targeting adolescent mental health in SSA have largely been implemented by non-specialist providers in public health facilities, schools, and community settings [10]. In Tanzania, an adolescent mental health intervention was developed through collaboration among adolescents, teachers, and researchers. This intervention included digital mood tracking, group discussions, sports, and art creation, alongside evidence-based topics addressing mental health issues, stigma, coping strategies, and mental health services [29]. The intervention demonstrated significant improvements, including a 16% increase in emotional literacy, 9% in prosocial behaviours, and knowledge gains related to mental health. Importantly, there was a 9.4% reduction in mental health difficulties among participants, highlighting the effectiveness of community co-created interventions in enhancing adolescent mental health.

Additionally, peer-delivered interventions have proven effective in enhancing adolescents’ access to and engagement with mental health services. Examples like the Youth Friendship Bench in Zimbabwe [30–33] and HERStory peer groups in South Africa [34] involved peers in designing and delivering interventions, leading to reductions in depression and anxiety symptoms, and improvements in health-related quality of life. The sessions helped adolescents process their emotions, manage stress, and cope with life challenges more effectively, which directly contributed to improved mental health. Qualitative evaluation evidence revealed that improvements in mental health were mainly attributed to the creation of safe and supportive spaces for adolescents to identify their most significant problems with a peer, with feelings of autonomy, active participation, and positive role modelling also identified as supportive of improved mental health [30–34].

The above interventions serve as examples of how mental health care can be delivered innovatively in low-resource settings. Sensitivity to contextual challenges is crucial for effective mental health care in communities. Existing interventions that have proven to be effective in SSA emphasize contextually and trauma-informed design [24,35,36–38], building referral networks to health clinics [36–39], and enhancing mental health literacy in families and communities [24,36,38,40]. These interventions have shown promising outcomes, including increased help-seeking behaviour and treatment adherence [24,36,37,39], reduced stigma [24,36,39,40], and improved mental health outcomes among adolescents [29–31,36; 38,41,42].

## Implications for global health and action

Addressing the mental health needs of adolescents in SSA requires a multi-level, coordinated approach based on the social-ecological framework for mental health and well-being [17]. By focusing on the individual, interpersonal, community, and societal levels, we can create a more integrated, inclusive, and sustainable mental health system. With the current treatment gap estimated at 90% [5] and predictions indicating a potential 130% increase in the burden of mental disorders in SSA by 2050 [43], increased funding and immediate action to establish healthier mental health pathways during adolescence are critical. With a clear focus on adolescence as a pivotal period for intervention, collaborative efforts across governments, communities, and international bodies can help bridge the mental health treatment gap and improve the well-being of future generations in SSA.

At the societal level, priority must be given to policies, governance, and public health systems. Despite the availability of strategies, interventions, and tools to assist governments in addressing adolescent mental health challenges, such as the Global Accelerated Action for the Health of Adolescents [44], the Mental Health Gap Action Program [45], and the Global Framework for Youth Mental Health [46], only 3 out of 48 SSA countries have a standalone adolescent mental health policy [47]. This disparity, compounded by underfunding, the shortage of mental health professionals, and limited healthcare access, severely hampers equitable and adequate mental health care. Governments, in collaboration with international bodies, must prioritize mental health policies, programs, and services by allocating adequate funding and resources. Interdisciplinary approaches involving public health experts, educators, psychologists, and social workers are essential in building a comprehensive system of support for adolescents. Such collaboration will ensure that mental health is integrated into broader health, education, and social service frameworks, thereby reinforcing its importance and implementation across sectors.

At the community level, we propose investing in community-based health strategies tailored to adolescent mental health in SSA. Guided by the WHO framework for meaningful engagement [48], this involves collaborating with adolescents living with mental health conditions and engaging key community stakeholders to understand barriers and gaps in mental health care delivery. Innovations in service delivery that are inclusive and contextually sensitive are crucial to creating environments where adolescents can access and benefit from mental health care and support. Co-creation and task-sharing approaches, involving communities and adolescents in the design and implementation of interventions, will not only help bridge the treatment gap but also empower adolescents to manage their mental health effectively within their local contexts, fostering resilience and well-being.

At the interpersonal level, capacity building and stigma reduction among healthcare workers, teachers, peers, and families are essential components of improving adolescent mental health. The social stigma surrounding mental illness in SSA can prevent adolescents from accessing care and sharing their struggles with family or friends. Peer pressure, social media influences, and limited peer support networks can further exacerbate mental health issues. Therefore, it is crucial to involve family members and peers in mental health education and support systems, ensuring they have the knowledge and tools to offer understanding and help reduce stigma. This can be achieved through education campaigns and training for healthcare workers, teachers, and families on how to recognize and respond to mental health issues in a supportive, non-judgmental way. Participatory approaches, where adolescents and community members actively contribute to the design and delivery of services, can foster greater acceptance and reduce stigma around mental health.

Addressing adolescent mental health challenges in SSA requires concerted global action, increased funding, and innovative, community-driven approaches. By prioritizing mental health policies and leveraging collaborative efforts across sectors, such as such as healthcare, education, and social services, we can build sustainable pathways to improve mental health outcomes for adolescents in the region. Community-based strategies, guided by meaningful engagement with adolescents and local stakeholders, are essential in bridging the current treatment gap. By co-creating contextually appropriate services and involving adolescents directly in the design and delivery of interventions, we can not only empower them to manage their mental health but also create more resilient and supportive communities.
